# Supplementary material for: Effects of 3′-Sialyllactose on Symptom Improvement in Patients with Knee Osteoarthritis: A Randomized Pilot Study
Source: Nutrients. 2024 Oct 8;16(19):3410. doi: 10.3390/nu16193410 (PMC11478649; doi:10.3390/nu16193410)
Supplement: Supplementary file 1 [file nutrients-16-03410-s001.zip › nutrients-3224507-supplementary.pdf]

No.	Age	Sex	Targeted site of the knee joint	group	Height (m)	Weight (kg)	BMI (kg/m2)	KL grade
1	66	2	2	3	1.572	66.2	26.789	2
2	45	2	1	2	1.639	56.8	21.144	1
3	47	2	1	1	1.629	62.3	23.477	2
4	51	2	1	3	1.547	54.0	22.564	1
5	51	2	1	2	1.558	52.9	21.793	2
6	57	2	1	1	1.611	65.1	25.084	1
7	52	2	1	2	1.532	59.7	25.436	2
8	54	2	2	3	1.509	61.2	26.877	2
9	57	2	2	3	1.594	56.3	22.158	1
10	59	2	2	1	1.555	49.0	20.264	1
11	54	1	2	2	1.694	63.2	22.024	2
12	54	1	1	3	1.681	73.2	25.905	3
13	53	1	1	1	1.712	72.7	24.804	2
14	53	1	2	2	1.657	74.9	27.280	1
15	54	1	2	3	1.649	64.4	23.683	3
16	49	1	2	2	1.649	67.7	24.897	2
17	45	1	1	3	1.767	69.4	22.227	2
18	48	1	1	1	1.769	73.0	23.327	1
19	48	2	2	2	1.637	60.6	22.614	2
20	50	2	2	3	1.623	59.8	22.702	2
21	50	2	1	1	1.627	85.0	32.110	2
22	45	1	2	2	1.681	56.4	19.959	2
23	43	1	1	3	1.662	70.8	25.631	2
24	43	1	1	1	1.856	92.2	26.765	2
25	60	1	2	2	1.622	60.0	22.806	2
26	60	1	2	3	1.613	67.4	25.905	2
27	58	1	1	1	1.781	85.2	26.860	2
28	57	1	1	2	1.640	64.4	23.944	2
29	47	2	2	2	1.560	70.2	28.846	2
30	46	2	2	3	1.636	54.2	20.250	2

No.	Age	Sex	Targeted site of the knee joint	group	Height (m)	Weight (kg)	BMI (kg/m2)	KL grade
31	60	1	1	2	1.718	70.4	23.852	2
32	54	1	2	1	1.700	67.6	23.391	2
33	63	1	1	3	1.775	80.1	25.424	1
34	53	1	2	3	1.676	60.5	21.538	3
35	54	1	1	2	1.651	62.4	22.892	2
36	53	1	2	1	1.632	63.4	23.804	2
37	51	1	2	2	1.660	79.4	28.814	1
38	50	1	2	3	1.793	106.9	33.252	2
39	70	2	2	3	1.500	54.8	24.356	3
40	56	2	1	1	1.453	57.4	27.188	2
41	62	2	1	2	1.666	70.8	25.508	2
42	56	2	1	1	1.540	50.5	21.294	2
43	71	2	1	2	1.465	53.2	24.788	2
44	76	2	1	3	1.648	76.0	27.983	4
45	66	2	2	1	1.499	62.7	27.904	2
46	52	1	1	3	1.662	66.8	24.183	1
47	48	1	1	1	1.650	78.0	28.650	2
48	48	1	2	2	1.675	85.8	30.581	2
49	51	2	1	1	1.564	57.0	23.302	2
50	53	1	2	3	1.697	70.3	24.411	2
51	50	1	2	1	1.708	73.3	25.126	2
52	54	1	1	1	1.603	63.2	24.595	2
53	46	2	2	1	1.670	67.3	24.131	2
54	54	2	2	2	1.561	62.9	25.813	2
55	62	1	2	1	1.638	73.4	27.357	2
56	59	2	2	2	1.520	51.7	22.377	2
57	55	2	1	1	1.510	57.2	25.087	1
58	82	2	1	3	1.430	55.4	27.092	4
59	60	2	1	3	1.533	59.3	25.233	2
60	51	2	1	2	1.590	67.4	26.660	2

Table S1. Characteristics of each population in this study. Sex: 1, male; 2, female. Targeted site of the knee joint: 1, right; 2, left. Group: 1, placebo; 2, 3′-SL 200 mg; 3, 3′-SL 600 mg. Abbreviations: BMI, body mass index; KL, Kellgren and Lawrence.

No.	VAS								
	Baseline			6week			12week		
	Placebo	3'-SL 200 mg	3'-SL 600 mg	Placebo	3'-SL 200 mg	3'-SL 600 mg	Placebo	3'-SL 200 mg	3'-SL 600 mg
1	40	20	50	40	20	50	20	10	30
2	70	50	40	70	20	60	30	30	50
3	50	50	40	30	30	40	30	30	10
4	60	30	20	30	10	20	10	10	30
5	20	60	40	10	50	10	30	20	10
6	60	30	20	40	20	10	20	10	0
7	30	50	40	20	20	60	10	30	40
8	50	20	30	50	20	5	50	20	10
9	40	10	30	30	10	30	10	10	10
10	20	50	30	20	30	0	10	20	0
11	10	20	30	20	20	40	15	20	50
12	30	40	20	50	30	no visite	40	40	no visite
13	50	10	70	40	10	30	20	10	50
14	30	70	30	50	40	30	40	0	40
15	30	50	40	30	20	20	no visite	10	0
16	30	50	50	30	50	50	20	20	20
17	20	30	60	20	40	30	30	30	20
18	10	20	50	20	10	30	20	10	30
19	20	30	90	40	10	no visite	10	0	no visite
20	10	30	30	30	20	30	no visite	30	10
average score	34.0	36.0	40.5	33.5	24.0	30.3	23.1	18.0	22.8

Table S2. VAS scores of each population in this study. Abbreviations: VAS, visual analog scale; 3′-SL, 3′-sialyllactose.

No.	KWOMAC								
	baseline			6week			12week		
	Placebo	3'-SL 200 mg	3'-SL 600 mg	Placebo	3'-SL 200 mg	3'-SL 600 mg	Placebo	3'-SL 200 mg	3'-SL 600 mg
1	31	39	39	54	30	53	26	26	34
2	44	39	31	44	35	22	24	32	28
3	37	32	33	27	34	24	24	21	28
4	37	35	34	36	31	26	32	23	24
5	39	41	41	34	32	32	41	31	26
6	40	49	35	33	46	30	29	40	28
7	56	50	42	43	38	45	32	31	40
8	52	33	34	51	35	27	42	31	27
9	36	38	41	27	36	39	20	28	35
10	43	40	47	33	37	25	30	27	23
11	57	49	58	58	30	45	46	30	32
12	45	56	33	48	20	no visite	48	46	no visite
13	59	40	54	37	32	19	31	33	39
14	53	56	42	76	39	32	61	24	37
15	51	54	37	53	44	39	no visite	32	32
16	50	55	54	48	62	32	45	58	24
17	38	60	56	38	58	51	32	59	53
18	35	34	41	39	29	29	48	29	21
19	52	42	94	60	33	no visite	52	24	no visite
20	34	49	50	30	48	51	no visite	66	30
average score	44.5	44.6	44.8	43.5	37.5	34.5	36.8	34.6	31.2

Table S3. KWOMAC scores of each population in this study. KWOMAC, Korean Western Ontario and McMaster Universities Osteoarthritis Index; 3′-SL, 3′-sialyllactose.
